# Phase 2 trial to assess lebrikizumab in patients with idiopathic pulmonary fibrosis

**DOI:** 10.1183/13993003.02442-2019

**Published:** 2021-02-04

**Authors:** Toby M. Maher, Ulrich Costabel, Marilyn K. Glassberg, Yasuhiro Kondoh, Takashi Ogura, Mary Beth Scholand, David Kardatzke, Monet Howard, Julie Olsson, Margaret Neighbors, Paula Belloni, Jeffrey J. Swigris

**Affiliations:** 1 NIHR Respiratory Clinical Research Facility, Royal Brompton Hospital and National Heart and Lung Institute, Imperial College London, London, UK; 2 Ruhrlandklinik, University of Duisburg-Essen, Essen, Germany; 3 University of Arizona, College of Medicine, Phoenix, AZ, USA; 4 Tosei General Hospital, Aichi, Japan; 5 Kanagawa Cardiovascular and Respiratory Center, Yokohama, Japan; 6 University of Utah, Salt Lake City, UT, USA; 7 Genentech, Inc., South San Francisco, CA, USA; 8 National Jewish Health, Denver, CO, USA

## Abstract

This phase 2, randomised, double-blind, placebo-controlled trial evaluated the efficacy and safety of lebrikizumab, an interleukin (IL)-13 monoclonal antibody, alone or with background pirfenidone therapy, in patients with idiopathic pulmonary fibrosis (IPF).

Patients with IPF aged ≥40 years with forced vital capacity (FVC) of 40%–100% predicted and diffusing capacity for carbon monoxide of 25%–90% predicted and who were treatment-naïve (cohort A) or receiving pirfenidone (2403 mg·day^−1^; cohort B) were randomised 1:1 to receive lebrikizumab 250 mg or placebo subcutaneously every 4 weeks. The primary endpoint was annualised rate of FVC % predicted decline over 52 weeks.

In cohort A, 154 patients were randomised to receive lebrikizumab (n=78) or placebo (n=76). In cohort B, 351 patients receiving pirfenidone were randomised to receive lebrikizumab (n=174) or placebo (n=177). Baseline demographics were balanced across treatment arms in both cohorts. The primary endpoint (annualised rate of FVC % predicted decline) was not met in cohort A (lebrikizumab *versus* placebo, −5.2% *versus* −6.2%; p=0.456) or cohort B (lebrikizumab *versus* placebo, −5.5% *versus* −6.0%; p=0.557). In cohort B, a non-statistically significant imbalance in mortality favouring combination therapy was observed (hazard ratio 0.42 (95% CI 0.17–1.04)). Pharmacodynamic biomarkers indicated lebrikizumab activity. The safety profile was consistent with that in previous studies of lebrikizumab and pirfenidone as monotherapies.

Lebrikizumab alone or with pirfenidone was not associated with reduced FVC % predicted decline over 52 weeks despite evidence of pharmacodynamic activity. Lebrikizumab was well tolerated with a favourable safety profile. These findings suggest that blocking IL-13 may not be sufficient to achieve a lung function benefit in patients with IPF.

## Abstract 2

**This phase 2 RCT found no benefit in FVC decline over 52 weeks in IPF patients for lebrikizumab *versus* placebo as monotherapy (n=78 *versus* 76) or in combination with pirfenidone (n=174 *versus* 177); pirfenidone therapy was consistent with previous results** https://bit.ly/313NVR8

## Introduction

Idiopathic pulmonary fibrosis (IPF) is a progressive, irreversible, fibrosing lung disease with an unpredictable rate of decline, a poor prognosis and a 10-year survival rate of ≤15% [1–3]. Pirfenidone is one of two approved antifibrotic therapies for IPF. Pirfenidone slows lung function decline as measured by % predicted forced vital capacity (FVC), improves progression-free survival (PFS) and reduces all-cause mortality [4–6]. However, little benefit has been shown for dyspnoea, quality of life or other clinically meaningful outcomes in the pivotal trials [4, 5]. As a result, there remains an unmet need for identifying new treatments that may offer additional clinical benefit to patients with IPF.

Interleukin (IL)-13 is a potent activator of fibroblasts, promoting extracellular matrix synthesis with potential pathogenic roles in fibrosis [7–10]. In mouse models, IL-13 deficiency or defective IL-13 signalling reduced lung fibrosis, whereas overexpression of IL-13 increased lung fibrosis [11–15]. In lung biopsy samples from patients with IPF, expression levels of IL-13, IL-13 receptors and IL-13 target genes were increased compared with normal controls [16, 17]. In bronchoalveolar lavage fluid from patients with IPF, IL-13 levels were elevated compared with normal controls, and IL-13 levels were negatively correlated with key measures of lung function, such as % predicted FVC and % predicted diffusing capacity for carbon monoxide (*D*_LCO_), suggesting pathogenic functions of IL-13 in patients with IPF [18]. C–C motif ligand 18 (CCL18) and periostin are IL-13 pathway biomarkers with levels that are elevated in IPF and are associated with lung function decline or death [19].

Lebrikizumab is a humanised monoclonal antibody that specifically binds soluble IL-13 to neutralise its activity and inhibit subsequent downstream signalling [20]. The RIFF study was designed initially to evaluate lebrikizumab as monotherapy and subsequent to the approval of pirfenidone, a second cohort study was added to evaluate lebrikizumab combination with pirfenidone for the treatment of patients with IPF.

## Methods

### Study design

RIFF (NCT01872689) was a randomised, multicentre, double-blind, placebo-controlled study of lebrikizumab *versus* placebo in patients with IPF. RIFF was initially designed as a time-to-event trial to assess the benefit of lebrikizumab on PFS. Sample size calculations, randomisation, blinding and dosing administration can be found in the supplementary material.

After the US Food and Drug Administration approved pirfenidone in October 2014, the RIFF protocol was amended in January 2015 to limit the number of patients (total 150 patients) and duration of blinded monotherapy assessment (52 weeks), designated as cohort A. Cohort B was added to assess the benefit of lebrikizumab *versus* placebo in patients receiving background pirfenidone therapy. The two cohorts were independent and enrolled sequentially. Patients entered a 28-day screening period after providing written informed consent.

**FIGURE 1:**
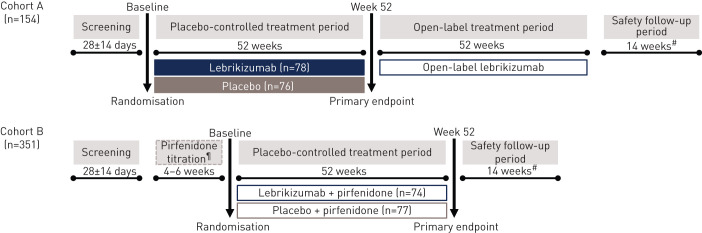
Study design. ^#^: Safety follow-up ended 18 weeks after the last dose of study drug; ^¶^: titration period allowed for patients who were pirfenidone-naïve at the time of enrolment.

In cohort A, patients were randomised 1:1 to receive lebrikizumab 250 mg monotherapy or placebo every 4 weeks for ≥52 weeks (figure 1). Study treatment was administered *via* subcutaneous injection, with the first injection occurring at randomisation (day 1, visit 2). After the placebo-controlled period, patients who did not discontinue received open-label lebrikizumab treatment for 52 weeks. All patients were followed for 18 weeks after last dose of study treatment (safety follow-up).

In cohort B, patients were randomised 1:1 to either lebrikizumab 250 mg or placebo every 4 weeks in combination with pirfenidone (≤2403 mg·day^−1^) for 52 weeks (figure 1). Pirfenidone-naïve patients initiated a run-in period (4–6 weeks) to allow pirfenidone titration to 2403 mg·day^−1^ (per prescribing information), the highest dose tolerated or recommended dose per country-specific guidelines [21]. Study treatment and safety follow-up matched those of cohort A.

This study was conducted in accordance with the principles of the Declaration of Helsinki and Good Clinical Practice. Approval was obtained from all institutional review boards prior to study initiation. Ethics approvals can be found in the supplementary material.

### Patients

Key eligibility criteria included age ≥40 years, diagnosis of IPF per the 2011 international guidelines ≤5 years before screening (confirmed by central review of high-resolution computed tomography during screening period or ≤12 months prior to screening, with multidisciplinary evaluation if needed), stable (<10% difference in FVC (L) between screening and randomisation), % predicted FVC ≥40% and ≤100%, % predicted *D*_LCO_ ≥25% and ≤90% and 6-min walk distance (6MWD) ≥100 m [3]. In cohort A, patients received no background IPF therapy for ≥4 weeks prior to randomisation and throughout the placebo-controlled period. In cohort B, patients received pirfenidone ≤2403 mg·day^−1^ for ≥4 weeks prior to randomisation and throughout the placebo-controlled period. Key exclusion criteria can be found in the supplementary material.

### Assessments

Serum chemokine CCL13, CCL18 and periostin, biomarkers of the IL-13 pathway, were measured at baseline and weeks 4, 24 and 52. In the prior development programme of lebrikizumab in asthma, antibodies against phospholipase B-like protein 2 (PLBL2), a process-related protein impurity, were detected in some patients; no association between immunogenicity and safety was observed [22]. Blood samples for detecting and characterising anti-drug antibodies (ADAs) to lebrikizumab and PLBL2 were collected at baseline and regular intervals to assess immunogenicity. Additional biomarker assessment and immunogenicity details can be found in the supplementary material.

Adverse events, serious adverse events (SAEs) and adverse events of special interest (AESIs) were assessed during the 52-week placebo-controlled period in both cohorts and during the lebrikizumab exposure period (from first dose of lebrikizumab until the end of safety follow-up) in cohort A. Adverse events were reported by the investigator and coded per Medical Dictionary for Regulatory Activities terms (version 20.1); no adjudication was performed. AESIs included anaphylactic reactions, local injection-site reactions, infections and malignancies. AESIs related to pirfenidone as identified in the phase 3 clinical trials included photosensitivity or rash, gastrointestinal-related adverse events (*e.g.* nausea, diarrhoea, vomiting) and elevated liver enzyme levels [4, 5].

### Endpoints and analyses

The primary endpoint was the annualised rate of decline in % predicted FVC through week 52 in the ITT population, which was compared across treatment arms with use of a random slope model at a 0.05 two-sided significance level. From the model, least squares means for the annualised rate of decline in each treatment arm included all FVC measurements collected at baseline, weeks 1, 4, 12, 24, 36, 44 and 52, and the difference between the two treatment arms was provided with 95% confidence intervals. No imputation for missing results because of death or other reasons were implemented. Missing data were handled by the model under the missing-at-random assumption (*i.e.* assuming that % predicted FVC decreases linearly over time).

Secondary efficacy endpoints in cohort A and cohort B included PFS, annualised rates of decline in % predicted *D*_LCO_ and 6MWD at week 52, annualised rate of change from baseline in A Tool to Assess the Quality of Life in IPF (ATAQ-IPF) total score at week 52 and time from randomisation to death from any cause [23]. The Borg Category Ratio 10 Scale (Borg scale) was collected as an exploratory endpoint. In cohort A, the Saint George's Respiratory Questionnaire was assessed. In cohort B, time-to-event endpoints were also evaluated, including time to respiratory-related hospitalisation, acute exacerbation or death, ≥10% absolute decline in % predicted FVC or death and ≥15% absolute decline in *D*_LCO_ or death. Kaplan–Meier estimates, log-rank tests stratified by baseline lung function for p values and Cox regression models to estimate hazard ratios and 95% confidence intervals were used to compare time-to-event endpoints.

All patients who received ≥1 dose of study drug and had ≥1 non-missing pharmacokinetic observation were included in the pharmacokinetic-evaluable population. Changes from baseline levels for pharmacodynamic biomarkers were evaluated in the ITT population.

## Results

### Patients

**FIGURE 2:**
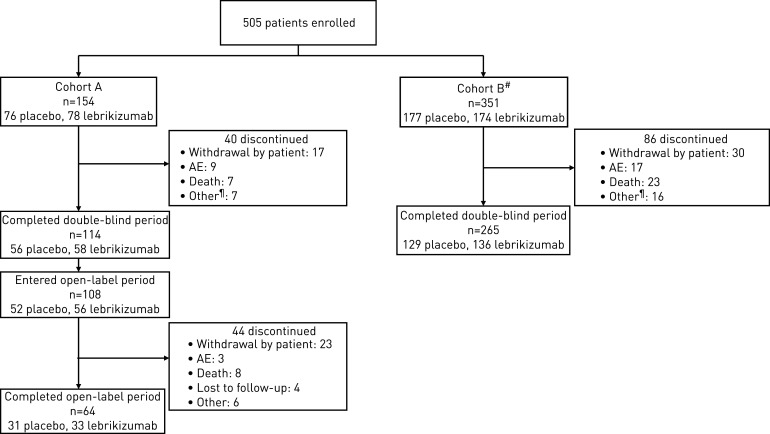
Patient disposition. AE: adverse event. ^#^: With background pirfenidone therapy; ^¶^: other reasons for discontinuation during the placebo-controlled period included lack of efficacy, lost to follow-up, physician decision and protocol violation.

In cohort A, 325 patients were screened at 156 sites in 13 countries (figure 2) between October 2012 and April 2015. Of 154 patients randomised (screen fail rate, 53%), 76 patients received placebo and 78 received lebrikizumab; 114 patients (74%) completed the placebo-controlled period. Of patients who completed the placebo-controlled period, 108 continued and received open-label lebrikizumab, and 64 of these patients (59%) completed the open-label period. In cohort B, 623 patients were screened between May 2015 and August 2016. Of 351 randomised receiving background pirfenidone therapy (screen fail rate, 44%), 174 patients received lebrikizumab and 177 received placebo; 265 patients (75%) completed the placebo-controlled period. The most common reasons for discontinuation in both cohorts were withdrawal by patient, death and adverse events.

The demographic profiles of the patients enrolled in the two treatment arms of cohort A and cohort B were comparable (table 1; supplementary table S1). The majority of patients were male (83% and 81% in cohorts A and B, respectively) and white (82% and 86%). The median (range) age was 70 (51–88) years in cohort A and 69 (50–86) years in cohort B.

**TABLE 1:** Baseline demographics and clinical characteristics

**Characteristic**	**Cohort A**			**Cohort B**		
	**Lebrikizumab (n=78)**	**Placebo (n=76)**	**All patients (N=154)**	**Lebrikizumab+pirfenidone (n=174)**	**Placebo+pirfenidone (n=177)**	**All patients (N=351)**
**Age years**	70 (51–88)	69 (52–84)	70 (51–88)	69 (52–85)	69 (50–86)	69 (50–86)
**Male n (%)**	65 (83.3)	63 (82.9)	128 (83.1)	137 (78.7)	147 (83.1)	284 (80.9)
**White n (%)**	66 (84.6)	60 (78.9)	126 (81.8)	151 (86.8)	149 (84.2)	300 (85.5)
**Time since diagnosis years**	0.92 (0.1–5.0)	1.53 (0.1–4.5)	1.07 (0.1–5.0)	1.51 (0.1–4.9)	1.54 (0.1–5.1)	1.54 (0.1–5.1)
**FEV_1_/FVC ratio**	0.80 (0.6–0.9)	0.81 (0.6–1.0)	0.81 (0.6–1.0)	0.81 (0.7–1.0)	0.82 (0.7–1.0)	0.82 (0.7–1.0)
**FVC % predicted**	73.0 (38.0–98.8)	72.8 (39.0–99.4)	73.0 (38.0–99.4)	71.5 (42.8–101.2)	73.4 (39.7–98.3)	72 (39.7–101.2)
***D*_LCO_ % predicted**	41.7 (22.3–73.6)	40.9 (25.0–67.9)	41.1 (22.3–73.6)	43.0 (12.9–71.3)	43.0 (19.5–84.0)	43.0 (12.9–84.0)
**ATAQ-IPF total score^#^**	70.5 (34–114)	69.0 (34–119)	70.0 (34–119)	71.0 (36–117)	70.0 (34–118)	70.0 (34–118)
**HRCT UIP diagnosis n (%)**						
n	78	75	153	174	177	348
Definite UIP	71 (91.0)	70 (93.3)	141 (92.6)	142 (81.6)	148 (83.6)	287 (82.6)
Possible UIP	7 (9.0)	5 (6.7)	12 (7.8)	28 (16.1)	28 (15.8)	56 (16.0)
Inconsistent with UIP	0	0	0	4 (2.3)	1 (0.6)	5 (1.4)
**Surgical biopsy n (%)**						
n	78	76	154	172	176	348
Definite UIP	31 (39.7)	24 (31.6)	55 (35.7)	61 (35.5)	54 (30.7)	115 (33.0)
Probable UIP	5 (6.4)	11 (14.5)	16 (10.4)	17 (9.9)	8 (4.5)	25 (7.2)
Possible UIP	0	0	0	0	0	0
Not UIP	0	0	0	0	0	0
NA	42 (53.8)	41 (53.9)	83 (53.9)	94 (54.7)	114 (64.8)	208 (59.8)

Data are presented as median (range), unless otherwise stated. All values are from measurements at baseline visit (day of randomisation); eligibility was based on measurements at screening visit. ^#^: range is 31–124. ATAQ-IPF: A Tool to Assess Quality of Life in IPF; *D*_LCO_: diffusing capacity of the lungs for carbon monoxide; FEV_1_: forced expiratory volume in 1 s; FVC: forced vital capacity; HRCT: high-resolution computed tomography; UIP: usual interstitial pneumonia.

Baseline disease characteristics, including % predicted FVC and % predicted *D*_LCO_, were comparable between treatment arms in both cohorts. Most patients (≥93%) had ≥1 medical history finding, including both active comorbid conditions and inactive past conditions. The most common targeted medical history conditions (≥20% in either arm) in cohort A were gastro-oesophageal reflux disease (GORD; lebrikizumab, 39.7% *versus* placebo, 42.1%), arthritis (19.2% *versus* 26.3%), coronary artery disease (29.5% *versus* 15.8%), type 2 diabetes mellitus (19.2% *versus* 25.0%) and pneumonia (20.5% *versus* 11.8%). In cohort B, the most common conditions were GORD (lebrikizumab, 52.3% *versus* placebo, 54.8%), coronary artery disease (24.1% *versus* 20.9%) and arthritis (19.5% *versus* 24.9%). Most patients (≥98%) in both treatment arms of both cohorts received ≥1 concomitant medication during the placebo-controlled period (supplementary table S2). The most common (≥50%) concomitant medications in any treatment arm were proton-pump inhibitors, statins, steroids and salicylates.

### Efficacy

**FIGURE 3:**
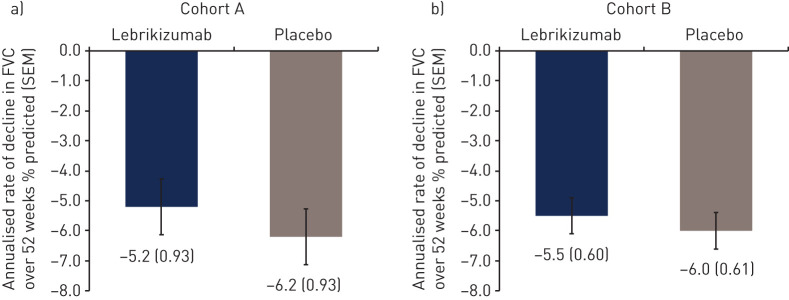
Annualised rate of decline in % predicted forced vital capacity (FVC) over 52 weeks. Cohort B was receiving background pirfenidone therapy.

The primary endpoint was not met for either cohort (figure 3). No difference was observed in the annualised rate±sem of decline in % predicted FVC over 52 weeks in either cohort A (lebrikizumab, −5.2±0.93% *versus* placebo, −6.2±0.93%; difference, 0.98±1.31%; p=0.456) or cohort B (lebrikizumab, −5.5±0.60% *versus* placebo, −6.0±0.61%; difference, 0.50±0.85%; p=0.557).

**FIGURE 4:**
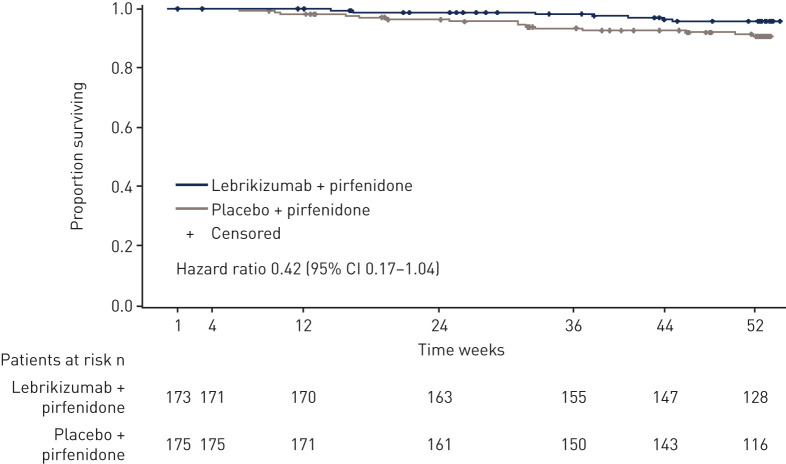
Time from randomisation to death from any cause in cohort B.

In cohort A, no effect on mortality was observed, with other secondary endpoints showing numerical trends that favoured lebrikizumab (table 2). A numerical imbalance in mortality favouring lebrikizumab plus pirfenidone treatment was observed in cohort B (HR 0.42 (95% CI 0.17–1.04); p=0.053) (figure 4). The small difference in event rate (seven deaths for lebrikizumab plus pirfenidone; 15 deaths for placebo plus pirfenidone) was driven by more deaths due to acute exacerbations in the placebo plus pirfenidone arm of cohort B. A similar trend was observed in favour of lebrikizumab for time to first acute exacerbation or death in cohort B (HR 0.61 (95% CI 0.29–1.25)) (supplementary figure S1). No treatment benefit was observed for other secondary endpoints in cohort B (table 2).

**TABLE 2:** Summary of secondary endpoints

	**Cohort A**			**Cohort B**		
	**Lebrikizumab (n=78)**	**Placebo (n=76)**	**p-value**	**Lebrikizumab+pirfenidone (n=174)**	**Placebo+pirfenidone (n=177)**	**p-value**
**Time-to-event analyses HR (95% CI)**						
PFS^#^	0.65 (0.39–1.09)		0.097^¶^	1.01 (0.72–1.42)		0.93^¶^
Time to first ≥10% absolute decline in % predicted FVC or all-cause mortality	0.79 (0.44–1.41)		0.42^¶^	0.84 (0.56–1.24)		0.37^¶^
Time to first ≥10% absolute decline in % predicted *D*_LCO_ or all-cause mortality	0.72 (0.23–2.26)		0.56^¶^	0.68 (0.37–1.23)		0.19^¶^
Time to first respiratory hospitalisation	NA		NA	0.89 (0.52–1.54)		0.68^¶^
Time to first acute IPF exacerbation or death from any cause	1.21 (0.41–3.61)		0.73^¶^	0.61 (0.29–1.25)		0.17^¶^
Time to first SGRQ worsening ≥7 or all-cause mortality	0.84 (0.54–1.31)		0.44^¶^	NA		NA
Time to all-cause mortality through week 52	1.01 (0.25–4.02)		0.99^¶^	0.42 (0.17–1.04)		0.053^¶^
**Annualised rate of decrease in % predicted *D*_LCO_ over 52 weeks**						
Slope	−4.24	−4.78	0.607	−5.57	−5.75	0.780
Absolute difference	0.54			0.18		
Relative difference %	11.3			3.2		
Decline ≥15% or death n (%)	1 (2)	3 (6)		3 (2.5)	5 (4.5)	
Relative difference %	−67.3			−44.9		
**Annualised rate of decline in 6MWD**						
Slope	−22.7	−44.6	0.312	−46.9	−25.6	0.203
Absolute difference	21.9			−21.4		
Relative difference %	49.1			−83.7		
**Annualised rate of decline in ATAQ-IPF total score over 52 weeks**						
Slope	4.78	6.89	0.385	5.45	5.61	0.905
Absolute difference	−2.10			−0.16		
Relative difference %	−30.5			−2.9		
**Proportion of patients with ≥10% decline in % predicted FVC or death from any cause**						
Decline ≥10% or death n (%)	9 (16.4)	12 (22.6)	0.89	22 (16.4)	19 (15.8)	0.67
Relative difference %	−27.7			3.7		

ATAQ-IPF: A Tool to Assess Quality of Life in IPF; *D*_LCO_: diffusing capacity of the lungs for carbon monoxide; FVC: forced vital capacity; HR: hazard ratio; IPF: idiopathic pulmonary fibrosis; PFS: progression-free survival; SGRQ: St. George's Respiratory Questionnaire; 6MWD: 6-min walk distance. ^#^: PFS was defined as time from randomisation to the first occurrence of: death from any cause, non-elective hospitalisation for any cause, relative decline in FVC ≥10%; ^¶^: log rank.

### Exploratory biomarker analyses

Baseline levels of serum CCL13, CCL18 and periostin were comparable between the arms within each cohort (supplementary table S3). Decreases in all three biomarkers were observed in the lebrikizumab-treated patients for both cohorts compared with their respective placebo or pirfenidone-treated controls, suggesting that lebrikizumab is active to block a component of these circulating biomarkers that is downstream of IL-13 (supplementary figure S2). The level of biomarker modulation between patients treated with lebrikizumab was consistent regardless of background pirfenidone treatment.

### Pharmacokinetics

Following subcutaneous administration of lebrikizumab 250 mg every 4 weeks with or without pirfenidone, the observed mean trough concentrations were similar at week 4 (supplementary table S4). The observed mean trough concentrations increased ≈2-fold from week 4 to week 52 for both cohorts. The mean±sd half-life estimates of lebrikizumab were similar across both cohorts (23.5±5.36 days for cohort A and 21.9±4.79 days for cohort B). Assessment of the exposure relationship to FVC change across 52 weeks in cohort B suggested the response was not strongly associated with exposure (supplementary figure S3). No exposure relationships were identified in patients that were hospitalised for a respiratory cause compared with those who were not, at weeks 24, 36 and 52 or for patients that later died.

### Safety

The duration of lebrikizumab treatment was similar between cohort A and cohort B (supplementary table S5). The total number of adverse events was comparable between treatment arms in both cohorts (table 3). During the placebo-controlled period, adverse events were reported in 75 (96.2%) and 71 (93.4%) patients in the lebrikizumab and placebo arms of cohort A, respectively, and 158 (90.8%) and 171 (96.6%) in the lebrikizumab and placebo arms of cohort B, respectively. The frequencies of common adverse events were generally similar between treatment arms in cohort A and were similar or lower in the lebrikizumab arm than in the placebo arm of cohort B (supplementary table S6). Photosensitivity reactions were less frequent in the lebrikizumab arm than in the placebo arm of cohort B (3.4% *versus* 12.4%, respectively). During the lebrikizumab exposure period, 96.9% of patients in cohort A experienced ≥1 adverse event (supplementary table S7).

**TABLE 3:** Overview of safety during the placebo-controlled period

**Event**	**Cohort A^¶^**			**Cohort B**		
	**Lebrikizumab (n=78)**	**Placebo (n=76)**	**All patients (N=154)**	**Lebrikizumab+pirfenidone (n=174)**	**Placebo+pirfenidone (n=177)**	**All patients (N=351)**
**Patients with ≥1 AE**	75 (96.2)	71 (93.4)	146 (94.8)	158 (90.8)	171 (96.6)	329 (93.7)
**Total AEs**	531	446	977	1013	1052	2065
**Deaths**	3 (3.8)	3 (3.9)	6 (3.9)	5 (2.9)	10 (5.6)	15 (4.3)
**Patients withdrawn from study due to AE**	5 (6.4)	9 (11.8)	14 (9.1)	16 (9.2)	27 (15.3)	43 (12.3)
**Patients with ≥1 AE with fatal outcome**	3 (3.8)	3 (3.9)	6 (3.9)	9 (5.2)	13 (7.3)	22 (6.3)
**Patients with ≥1 SAE**	23 (29.5)	19 (25.0)	42 (27.3)	56 (32.2)	47 (26.6)	103 (29.3)
Patients with ≥1 SAE leading to withdrawal from treatment	6 (7.7)	6 (7.9)	12 (7.8)	12 (6.9)	14 (7.9)	26 (7.4)
Patients with ≥1 SAE leading to dose modification/interruption	5 (6.4)	3 (3.9)	8 (5.2)	6 (3.4)	4 (2.3)	10 (2.8)
Patients with ≥1 treatment-related SAE	2 (2.6)	1 (1.3)	3 (1.9)	3 (1.7)	6 (3.4)	9 (2.6)
**Patients with ≥1 AE leading to withdrawal from treatment**	8 (10.3)	9 (11.8)	17 (11.0)	20 (11.5)	26 (14.7)	46 (13.1)
**Patients with ≥1 AE leading to dose modification/interruption**	7 (9.0)	5 (6.6)	12 (7.8)	11 (6.3)	11 (6.2)	22 (6.3)
**Patients with ≥1 treatment-related AE**	25 (32.1)	19 (25.0)	44 (28.6)	30 (17.2)	30 (16.9)	60 (17.1)
Patients with ≥1 treatment-related AE leading to withdrawal from treatment	0	2 (2.6)	2 (1.3)	3 (1.7)	8 (4.5)	11 (3.1)
Patients with ≥1 treatment-related AE leading to dose modification/interruption	2 (2.6)	0	2 (1.3)	1 (0.6)	1 (0.6)	2 (0.6)
**AESIs**						
Injection-site reactions	13 (16.7)	6 (7.9)	19 (12.3)	5 (2.9)	5 (2.8)	10 (2.8)
Infections (broad)	51 (65.4)	41 (53.9)	92 (59.7)	106 (60.9)	114 (64.4)	220 (62.7)
Infections (narrow)	0	0	0	0	1 (0.6)	1 (0.3)
Malignancies^#^	5 (6.4)	2 (2.6)	7 (4.5)	13 (7.5)	12 (6.8)	25 (7.1)
AEs related to pirfenidone	5 (6.4)	3 (3.9)	8 (5.2)	64 (36.8)	70 (39.5)	134 (38.2)
AEs leading to withdrawal from pirfenidone	1 (1.3)	1 (1.3)	2 (1.3)	9 (5.2)	12 (6.8)	21 (6.0)

Data are presented as n (%) or n. AE: adverse event; AESI: adverse event of special interest; NA: not available; SAE: serious adverse event. ^#^: Identified with Standardised Medical Dictionary for Regulatory Activities (MedDRA) Queries “narrow”; ^¶^: patients were not required to stop study drug to be treated with pirfenidone.

In the placebo-controlled period, 23 (29.5%) and 19 (25.0%) patients reported SAEs in the lebrikizumab and placebo arms of cohort A, respectively; 56 (32.2%) and 47 (26.6%) reported SAEs in the lebrikizumab and placebo arms of cohort B, respectively (table 3). Treatment-related SAEs (investigator assessment) were reported in two patients who received lebrikizumab and one who received placebo in cohort A and three who received lebrikizumab and six who received placebo in cohort B with background pirfenidone. The most common SAEs (≥2% of patients in either arm in either cohort) were IPF (worsening or exacerbation as reported by the investigator), respiratory tract infection and pneumonia. In cohort A, pneumothorax, pulmonary embolism and cardiac failure SAEs also occurred in ≥2% of patients (in either arm). During the lebrikizumab exposure period, 57 patients (43.8%) in cohort A experienced a total of 92 SAEs, of which three had treatment-related SAEs as assessed by the investigator. Incidences of AESIs were comparable between treatment arms in both cohorts (table 3). No adverse events met Sampson's criteria for anaphylaxis.

In cohort A, 19 patients died during the study; 15 deaths occurred during the lebrikizumab exposure period (supplementary table S8). During the placebo-controlled period, three patients (3.8%) in the lebrikizumab arm and three (3.9%) in the placebo arm died. Nine deaths occurred during open-label lebrikizumab treatment and four during safety-follow-up. The most common cause of death in cohort A was IPF (10 patients). Three deaths in cohort A were considered related to study drug by the investigator: IPF (during safety follow-up following double-blind placebo treatment), acute respiratory failure (5 days after receiving open-label lebrikizumab following double-blind placebo treatment) and pulmonary embolism (during the placebo-controlled period following lebrikizumab treatment).

In cohort B, 29 patients died; 22 deaths occurred during the placebo-controlled period: nine (5.2%) and 13 (7.3%) in the lebrikizumab and placebo arms, respectively (supplementary table S8). Seven deaths in cohort B occurred during safety-follow-up. IPF was the most common cause of death in both lebrikizumab and placebo arms (five and eight patients, respectively). Of 29 deaths in cohort B, one in the placebo arm with background pirfenidone (due to IPF, during the placebo-controlled period) was considered related to study drug by the investigator.

Lebrikizumab ADAs and PLBL2 antibodies were detected in 5.7% (14 of 247) and 24.7% (42 of 170), respectively, of lebrikizumab-treated patients in both cohorts. The median time to onset of lebrikizumab ADAs was ∼12 weeks. There was no evidence to suggest an adverse impact the presence of positive anti-lebrikizumab antibodies or anti-PLB2 antibodies on safety profiles of patients who received lebrikizumab treatment (data not shown).

## Discussion

RIFF failed to meet the primary endpoint in patients with IPF. Lebrikizumab monotherapy (cohort A) was not associated with a treatment benefit on lung function, mortality or other patient-relevant outcomes. Addition of lebrikizumab to background pirfenidone therapy (cohort B) was also not associated with a treatment benefit on lung function. The overall rates of FVC decline observed in cohorts A and B were similar despite the use of background pirfenidone in cohort B. However, direct comparisons cannot be made as they were conducted as separate studies in different trial eras under a single protocol. In cohort B, a non-statistically significant imbalance in mortality favouring combination therapy was observed (HR 0.42 (95% CI 0.17–1.04)). The difference in event rate was driven by more deaths due to acute exacerbations in patients who received placebo with background pirfenidone. A similar trend favouring lebrikizumab was observed for time to acute exacerbation or death in cohort B (HR 0.61 (95% CI 0.29–1.25)). Reduced mortality is an important outcome in IPF. In the phase 3 trials of pirfenidone and nintedanib, which led to their approval to treat IPF, the effect on mortality was reported across individual studies and in pooled analyses combining phase 3 data for each therapy [4–6, 24, 25]. A mortality benefit was not observed for nintedanib in either individual trial nor in the pooled analysis [24, 25]. In the pirfenidone phase 3 studies, a mortality benefit was observed in one out of three phase 3 trials and in the pooled analysis [4–6]. Thus, the observed limited reduction in the rate of acute exacerbations and subsequent death of patients treated with combination therapy may warrant further investigation, as this study was powered to show only lung function changes.

Other clinical trials in IPF that have assessed IL-13 antibodies have also demonstrated a lack of efficacy in this patient population, suggesting that IL-13 may not be an appropriate therapeutic target in IPF. A phase 2 randomised, double-blind, placebo-controlled trial that assessed the safety, tolerability and change in FVC at 52 weeks of the anti–IL-13 QAX576 in 60 patients with IPF was terminated [26]. A phase 2, randomised, double-blind, placebo-controlled trial that assessed change in % predicted FVC at 72 weeks of the anti–IL-13 tralokinumab in 302 patients with IPF was terminated due to lack of efficacy after the interim analysis [27]. A phase 2, randomised, double-blind, placebo-controlled trial that assessed change in % predicted FVC at 52 weeks of the anti–IL-13/IL-4 SAR156597 in 325 patients with IPF showed no significant difference in the primary efficacy endpoint [28]. However, a similar trend towards a decrease in acute exacerbations was reported in cohort B. The reported rates of acute exacerbations in the IPF clinical trial setting is variable (4–28%) likely due to differences in study population, acute exacerbation definition and statistical methods [29]. In cohort B, the event rate in the lebrikizumab plus pirfenidone arm was 2.9% (five acute exacerbations) *versus* 6.2% (11) in placebo plus pirfenidone arm (HR 0.45 (0.16–1.31); p=0.1346). Although the overall number of acute exacerbations was low and the difference in the rate of acute exacerbations did not reach statistical significance, it is interesting to note that there were no deaths associated with acute exacerbations in the lebrikizumab plus pirfenidone arm, while seven of 11 patients in the placebo plus pirfenidone arm died, consistent with reported rates of mortality post-acute exacerbation ≥50%. While these findings are similar to results reported in the SAR15697 trial targeting IL-4 and IL-13, there was no evidence of a treatment difference between lebrikizumab and placebo in cohort A (HR 1.21 (0.41–3.61); p=0.73).

The pharmacokinetics (*e.g.* half-life estimates) and pharmacodynamics of lebrikizumab were similar in both cohorts, suggesting that background pirfenidone therapy did not impact lebrikizumab concentrations or activity. Pharmacodynamic measures supported lebrikizumab biological activity, both alone and in the presence of background pirfenidone therapy. Furthermore, efficacy/response relationships were generally flat across endpoints and did not suggest that increased dosing would have led to greater improvements in efficacy endpoints.

This study has several limitations. Each independent cohort was powered to detect differences in lung function between lebrikizumab and placebo treatment arms, but not differences in other outcomes. The approval of two antifibrotic therapies in late 2014 contributed to early patient discontinuation in cohort A, which limited the amount of data captured to evaluate lebrikizumab as monotherapy. During the recruitment phase of cohort A, 168 patients (51.6%) screened did not meet eligibility criteria similar to other anti-IL-13 trials executed between 2012 and 2016. It has been suggested that many of the patients referred to the anti-IL-13 trials may have been screening failures from the large phase 3 trials completing at that time and thus may not reflect the real-world population or populations studied in the prior phase 3 trials. In contrast, FVC and *D*_LCO_ measurements used standardised equipment, training and efforts with central over-read; thus, standardisation issues did not likely contribute to the lack of observed effect.

Lebrikizumab as monotherapy and in combination with pirfenidone was well tolerated, with a favourable safety profile, consistent with those reported for lebrikizumab and pirfenidone individually in phase 2 and 3 clinical trials [4, 5, 30, 31]. The incidence of lebrikizumab or PLBL2 antibodies in both lebrikizumab- and placebo-treated patients did not appear to affect pharmacokinetics or safety.

In conclusion, lebrikizumab monotherapy or in combination with background pirfenidone therapy did not reduce lung function decline or provide other clinically significant benefits in patients with IPF.

## Supplementary material

10.1183/13993003.02442-2019.Supp1 **Please note:** supplementary material is not edited by the Editorial Office, and is uploaded as it has been supplied by the author.
Supplementary material ERJ-02442-2019.SUPPLEMENT

## Shareable PDF

10.1183/13993003.02442-2019.Shareable1 This one-page PDF can be shared freely online.
Shareable PDF ERJ-02442-2019.Shareable
