# Supplementary material for: Phase 2 trial to assess lebrikizumab in patients with idiopathic pulmonary fibrosis
Source: Eur Respir J. 2021 Feb 4;57(2):1902442. doi: 10.1183/13993003.02442-2019 (PMC7859504; doi:10.1183/13993003.02442-2019)
Supplement: Supplementary file 1 [file ERJ-02442-2019.SUPPLEMENT.pdf]

SUPPLEMENT:

Phase 2 trial to assess lebrikizumab in patients with idiopathic pulmonary fibrosis

Toby M. Maher, Ulrich Costabel, Marilyn K. Glassberg, Yasuhiro Kondoh, Takashi Ogura, Mary Beth Scholand, David Kardatzke, Monet Howard, Julie Olsson, Margaret Neighbors, Paula Belloni, Jeffrey J. Swigris

This supplement contains:

- Supplemental methods
- 3 supplemental figures
- 10 supplemental tables

Supplemental Methods

Ethics approvals

All participants provided informed, written consent. Approval of the study protocol was obtained from the institutional review board or independent ethics committee at each study site prior to study initiation. (See Supplemental table S9 for a full list of investigators and ethics approvals.)

Determination of sample size

The study was initially designed as a time-to-event trial to assess the benefit of lebrikizumab as monotherapy on progression-free survival (PFS). PFS was defined as time from randomisation to the first occurrence of: death from any cause, non-elective hospitalization for any cause, relative decline in FVC (L) ≥10%. After the approval of antifibrotic therapy to treat IPF in October 2014, the protocol was amended in January 2015 to add Cohort B to assess lebrikizumab vs. placebo with background pirfenidone. When the protocol was amended, the primary endpoint for both cohorts was changed from PFS to annualised rate of decline in % predicted forced vital capacity (FVC) through Week 52.

In Cohort A, it was estimated that a sample size of 75 patients in each treatment group would be needed to achieve ≈80% power to detect a 3.7% difference in the annualised rate of decline in % predicted FVC over 52 weeks. This is assuming a common SD of 8% (as reported in the placebo group of ASCEND) using a 2-group *t* test with a 0.05 two-sided significance level.

In Cohort B, it was estimated that a sample size of 165 patients in each treatment group would be needed to achieve ≈80% power to detect a 2.5% difference in the annualised rate of decline in % predicted FVC over 52 weeks. A similar assumption for the SD applied here.

Exclusion criteria

Key exclusion criteria included:

- History of severe allergic reaction or anaphylactic reaction to a biologic agent or known hypersensitivity
- Evidence of other known causes of interstitial lung disease or clinically significant lung disease other than IPF
- Lung transplant expected within 12 months of screening
- Post-bronchodilator forced expiratory volume in 1 second (FEV_1)/FVC ratio <0.7
- Positive bronchodilator response indicated by an increase of ≥12% predicted and 200 mL increase in either FEV_1 or FVC
- Hospitalisation due to IPF exacerbation ≤4 weeks prior to or during screening
- Cardiac or liver disease, known current malignancy, infections or immunodeficiency
- Chronic oral corticosteroid therapy

Randomisation and blinding

Patients were randomised via an interactive voice/Web-based response system (IxRS). Within each cohort, dynamic hierarchical randomisation was performed centrally and stratified by the following:

- Region: United States, Europe/Canada, other
- Lung function: % predicted FVC <50%, 50% to 75%, <75%
- Serum periostin concentration: <50 ng/mL, ≥50 ng/mL

Patients, all study site personnel and the Sponsor were blinded to the treatment assignment throughout the placebo-controlled period. Treatment for each cohort was unblinded at the time of the primary analysis.

Dosing and administration

Lebrikizumab

All patients in Cohorts A and B received lebrikizumab 250 mg or placebo every 4 weeks for the 52-week placebo-controlled period. All patients received a total of 2 injections per dosing visit for a minimum of 13 doses of study treatment during the placebo-controlled, blinded, study treatment period. Patients in Cohort A who experienced confirmed disease progression, defined as ≥10% decline in FVC (mL/year, relative change) or non-elective hospitalisation, during the placebo-controlled period could initiate rescue therapy, including use of pirfenidone or nintedanib, at the investigator's discretion if approved by local regulatory authorities (see Supplemental table S10). During the open-label period, patients in Cohort A could add treatment with pirfenidone, nintedanib or other regionally approved IPF therapies at the investigator's discretion.

Pirfenidone

The recommended dose of pirfenidone is 2403 mg/day (or 1800 mg/day for patients in Japan) administered in divided doses 3 times per day (TID) with food. Patients in Cohort B who entered screening receiving stable pirfenidone were randomised to receive lebrikizumab or placebo. Patients in Cohort B who were treatment-naive to pirfenidone initiated titration during the run-in period over 14 days, as tolerated, to the full dose of 9 capsules per day (3 capsules TID) as follows:

- Days 1–7: 1 capsule TID
- Days 8–14: 2 capsules TID
- Day 15 and onward: 3 capsules TID

Combination treatment with pirfenidone and nintedanib was not permitted.

Biomarker assessments

CCL13 was measured using the R&D Systems Quantikine human CCL13-MCP-4 immunoassay (DCC130) with lower limit of quantification (LLOQ) of 31.2 pg/mL. CCL18 was measured using the IMPACTD R-CID10.01 chip with LLOQ of 6 ng/mL. Periostin was measured using the Elecys assay on a Cobas e601 instrument with LLOQ of 10 ng/mL.

Immunogenicity

Lebrikizumab ADAs and PLBL2 antibodies were tested using a validated immunoassay by Covance Laboratories, Inc. (Chantilly, VA). The assay had a relative sensitivity 64 ng/mL of a surrogate positive control, a monoclonal anti-idiotypic antibody against lebrikizumab and 133 ng/mL of a surrogate positive control for monoclonal antibody against PLBL2. The assay could detect 500 ng/mL of this surrogate positive control in the presence of 50 µg/mL lebrikizumab or 250 ng/mL of this surrogate positive control in the presence of 200 ng/mL PLBL2.

A positive ADA sample was defined as one in which the presence of detectable ADAs could be confirmed by competitive binding with lebrikizumab. Treatment-induced ADA patients were those with negative or missing baseline ADA results and ≥1 positive post-baseline ADA result. Treatment-enhanced ADA patients were those with positive ADA results at baseline who then had ≥1 post-baseline titre result that were ≥0.6 more titre units than the baseline titre units. The baseline prevalence and post-baseline incidence of ADAs were calculated from the number of patients who tested ADA positive at baseline or post-baseline divided by either the total number of patients with evaluable samples at the baseline timepoint or post-baseline, respectively. Similar rules were used to define PLBL2 antibodies.

Supplemental Figures

SUPPLEMENTAL FIGURE S1. Time from randomisation to first acute exacerbation or death in Cohort B

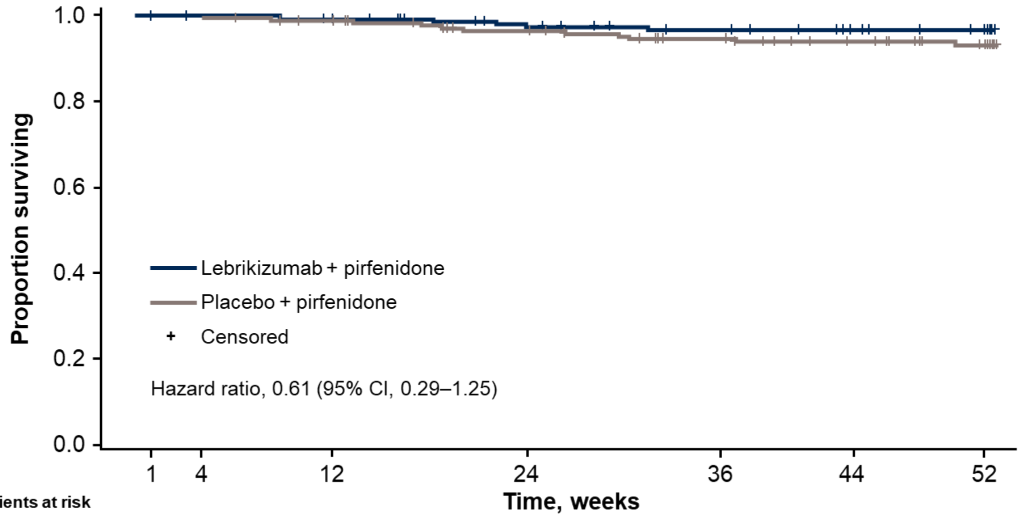

	1	4	12	24	36	44	52
**No. of patients at risk**							
Lebrikizumab + pirfenidone	172	170	168	159	152	144	125
Placebo + pirfenidone	175	174	170	159	148	140	113
**Cumulative no. of events by visit**							
Lebrikizumab + pirfenidone	0	0	3	6	9	12	12 (6.9%)
Placebo + pirfenidone	0	2	7	12	16	17	19 (10.9%)

SUPPLEMENTAL FIGURE S2. Pharmacodynamics of biomarkers for lebrikizumab target engagement

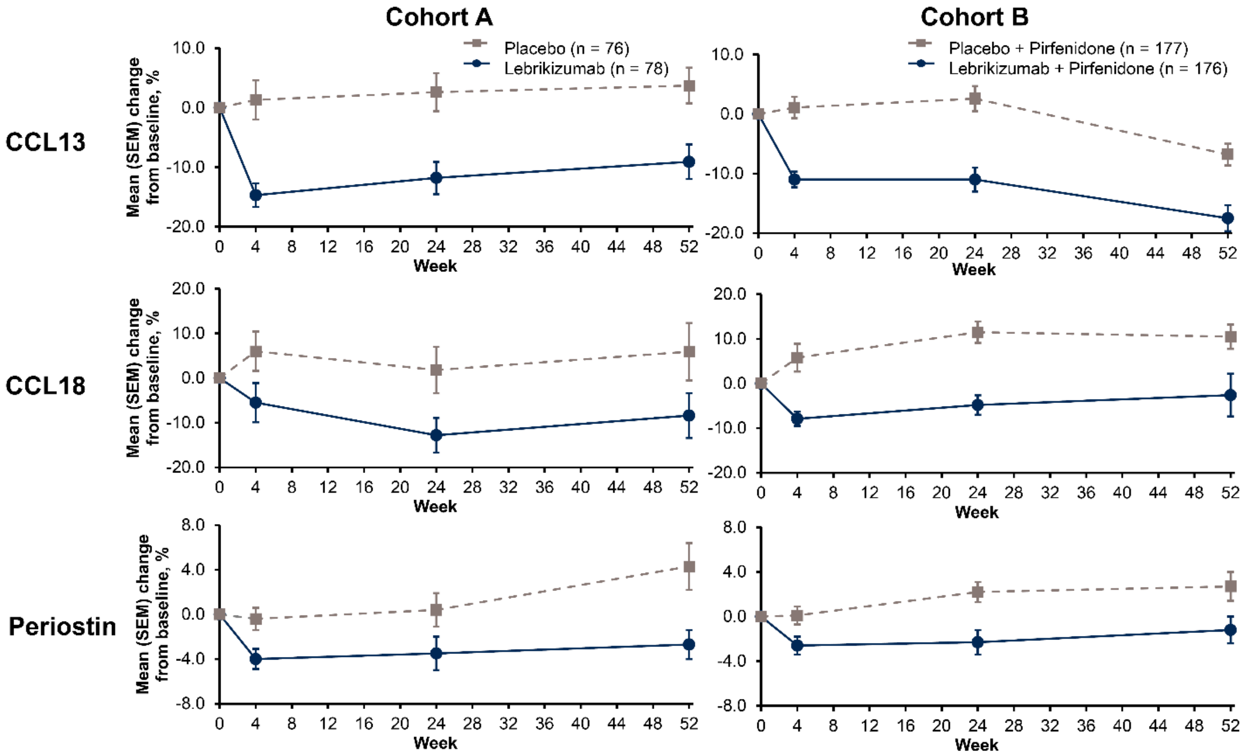

CCL: chemokine C-C motif ligand.

SUPPLEMENTAL FIGURE S3. Relationship between lebrikizumab exposure and FVC change at Week 52

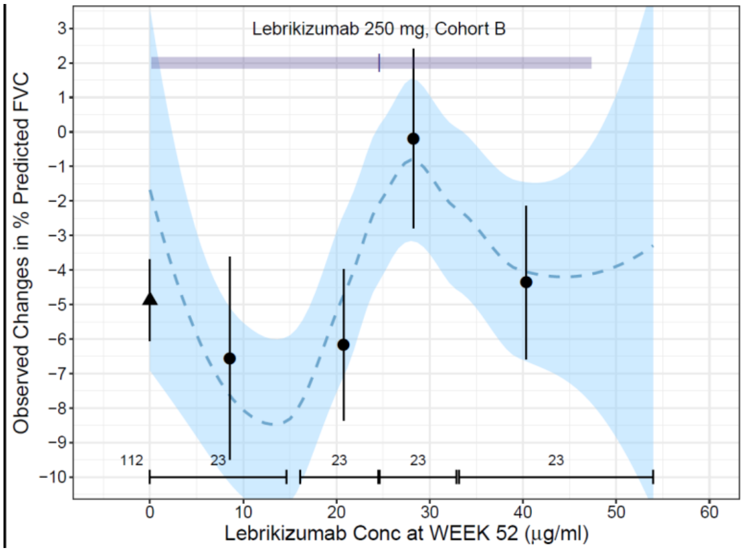

FVC: forced vital capacity.

Supplemental Tables

SUPPLEMENTAL TABLE S1. Baseline 6MWD data

Characteristic[#]	Cohort A			Cohort B		
	**Lebrikizumab (n=78)**	**Placebo (n=76)**	**All patients (N=154)**	**Lebrikizumab + pirfenidone (n=174)**	**Placebo + pirfenidone (n=177)**	**All patients (N=351)**
6MWD, m	436.5 (150.0–926.0)	455.5 (190.0–975.0)	450.0(150.0–975.0)	455 (156.0–996.3)	510 (118.0–9900)	480 (118.0–996.3)
Difference in Borg scale[+]	1.5 (−2.0–9.5)	2.0 (−1.0–10.0)	2.0 (−2.0–10.0)	2.0 (−2.7–9.5)	2.0 (−2.5–9.5)	2.0 (−2.7–9.5)

6MWD, 6-minute walk distance.

[#] Values are median (range) unless otherwise noted. All values are from measurements at baseline visit (day of randomisation); eligibility was based on measurements at screening visit.

[¶] Results may not be reliable due to inconsistency with reporting and measurement between study sites.

[+] Baseline difference in Borg scale represents change in dyspnoea after the 6MWD test compared with before the test. The Borg scale range is 0–10.

SUPPLEMENTAL TABLE S2. Concomitant medications reported during the placebo-controlled period

Patients reporting concomitant medication use, n (%)[#]	Cohort A			Cohort B		
	Lebrikizumab (n=78)	Placebo (n=76)	All patients (n=154)	Lebrikizumab + pirfenidone (n=174)	Placebo + pirfenidone (n=177)	All patients (n=351)
≥ 1 concomitant medication	77 (98.7)	76 (100.0)	153 (99.4)	174 (100.0)	174 (98.3)	348 (99.1)
Medication use by class[¶]						
Proton pump inhibitors	52 (66.7)	44 (57.9)	96 (62.3)	125 (71.8)	122 (68.9)	247 (70.4)
Statins	41 (52.6)	35 (46.1)	76 (49.4)	86 (49.4)	85 (48.0)	171 (48.7)
Steroids	27 (34.6)	28 (36.8)	55 (35.7)	93 (53.4)	98 (55.4)	191 (54.4)
Salicylates	36 (46.2)	34 (44.7)	70 (45.5)	78 (44.8)	88 (49.7)	166 (47.3)
Antihistamines	21 (26.9)	20 (26.3)	41 (26.6)	59 (33.9)	73 (41.2)	132 (37.6)
Analgesics	26 (33.3)	28 (36.8)	54 (35.1)	50 (28.7)	66 (37.3)	116 (33.0)
Herbal, homeopathic and dietary supplements	32 (41.0)	24 (31.6)	56 (36.4)	51 (29.3)	60 (33.9)	111 (31.6)
Non-steroidal anti-inflammatory drugs	26 (33.3)	30 (39.5)	56 (36.4)	52 (29.9)	54 (30.5)	106 (30.2)
Cough preparations	23 (29.5)	15 (19.7)	38 (24.7)	48 (27.6)	63 (35.6)	111 (31.6)
Vaccines, toxoids and serologic agents	25 (32.1)	25 (32.9)	50 (32.5)	41 (23.6)	52 (29.4)	93 (26.5)
Macrolide antibiotics	17 (21.8)	15 (19.7)	32 (20.8)	50 (28.7)	46 (26.0)	96 (27.4)
β-adrenoceptor blocking agents	23 (29.5)	15 (19.7)	38 (24.7)	43 (24.7)	47 (26.6)	90 (25.6)
Bronchodilators and anti-asthmatic drugs	13 (16.7)	15 (19.7)	28 (18.2)	38 (21.8)	56 (31.6)	94 (26.8)
Angiotensin II receptor antagonists	22 (28.2)	19 (25.0)	41 (26.6)	37 (21.3)	38 (21.5)	75 (21.4)
Penicillins	17 (21.8)	10 (13.2)	27 (17.5)	27 (15.5)	54 (30.5)	81 (23.1)
Quinolone antibiotics	17 (21.8)	14 (18.4)	31 (20.1)	37 (21.3)	32 (18.1)	69 (19.7)

[#] Concomitant medication use occurred at any time from first dose of study treatment through the end of the placebo-controlled period.

¶ Concomitant medication classes reported in ≥ 20% of patients in either Cohort A or Cohort B are shown. When pirfenidone was excluded as a concomitant medication, < 1% and 2% of patients in Cohort A and Cohort B, respectively, received immunosuppressants. Medications were coded using Medical Dictionary for Regulatory Activities (MedDRA) version 20.1.

SUPPLEMENTAL TABLE S3. Baseline biomarker levels

Biomarker#	Cohort A			Cohort B		
	**Lebrikizumab (n=78)**	**Placebo (n=76)**	**All patients (N=154)**	**Lebrikizumab + pirfenidone (n=174)**	**Placebo + pirfenidone (n=177)**	**All patients (N=351)**
CCL13, pg/mL	264.0 (215.2–324.7)	270.9 (222.4–339.4)	270.5 (219.8–333.9)	302.0 (225.6, 381.7)	295.2 (244.2–383.7)	296.9 (229.3–382.1)
CCL18, ng/mL	628.6 (399.7–873.8)	659.9 (341.5–900.6)	657.5 (360.5–882.3)	269.8 (205.0–357.7)	275.0 (217.5–374.6)	273.5 (211.3–363.2)
Periostin, ng/mL	64.4 (54.7–81.0)	66.9 (57.2–80.7)	65.6 (56.1–80.9)	62.2 (54.9–74.1)	64.8 (53.0–79.0)	63.5 (53.4–75.4)

CCL: chemokine C-C motif ligand.

Values are median (interquartile range).

SUPPLEMENTAL TABLE S4. Summary of pharmacokinetics following lebrikizumab administration

Cohort	Treatment	Summary	Mean (SD)					
			Week 4 $C_{min,}$ µg/mL	Week 12 $C_{min,}$ µg/mL	Week 24 $C_{min,}$ µg/mL	Week 36 $C_{min,}$ µg/mL	Week 52 $C_{min,}$ µg/mL	$t_{1/2}$, days
A	Lebrikizumab 250 mg	N	74	68	65	2	61	35
		Mean (SD)	14.0 (4.86)	24.4 (9.86)	28.5 (12.5)	29.6 (14.1)	28.5 (14.0)	23.5 (5.36)
B	Lebrikizumab 250 mg + pirfenidone	N	170	165	153	146	137	125
		Mean (SD)	14.9 (5.75	25.1 (11.0)	25.7 (12.4)	25.6(13.8)	25.2(12.7	21.9 (4.79)

C_{min}: observed minimum serum concentration; $t_{1/2}$: elimination half-life.

SUPPLEMENTAL TABLE S5. Treatment exposure

	Cohort A			Cohort B		
	**Lebrikizumab (n=78)**	**Placebo (n=76)**	**All patients (N=154)**	**Lebrikizumab + pirfenidone (n=174)**	**Placebo + pirfenidone (n=177)**	**All patients (N=351)**
**Lebrikizumab or placebo exposure during the placebo-controlled period**						
Treatment duration, mean (SD), weeks	44.7 (18.3)	44.9 (18.0)	44.8 (18.1)	42.4 (13.1)	41.3 (13.1)	41.8 (13.1)
No. of doses, mean (SD)	12.0 (4.5)	12.1 (4.4)	12.0 (4.5)	11.4 (3.3)	11.2 (3.3)	11.3 (3.3)
**Lebrikizumab exposure period**[#]						
	**Lebrikizumab to lebrikizumab (n=78)**	**Placebo to lebrikizumab (n=52)**	**All patients (N=130)**			
Treatment duration, mean (SD), weeks	79.1 (38.6)	39.3(15.0)	63.2 (36.9)	–	–	–
No. of doses, mean (SD)	20.5 (9.6)	10.8 (3.8)	16.6 (9.1)	–	–	–
**Pirfenidone exposure during the placebo-controlled period**						
Treatment duration, mean (SD), weeks	–	–	–	46.7 (12.7)	44.9 (13.4)	45.8 (13.1)
Daily dose, mean (SD), mg	–	–	–	2088.6 (470.6)	2053.4 (504.2)	2070.9 (487.5)

[#] The lebrikizumab exposure period included all adverse events reported from the first dose of study drug until the end of safety follow-up. This included patients who were randomised to receive lebrikizumab and all patients who received lebrikizumab during the open-label treatment period (including patients who were randomised to receive placebo).

SUPPLEMENTAL TABLE S6. Summary of AEs by preferred terms in ≥10% of patients in any treatment arm during the placebo-controlled period

**AE**	**Cohort A**		**Cohort B**	
	**Lebrikizumab (n=78)**	**Placebo (n=76)**	**Lebrikizumab + pirfenidone (n=174)**	**Placebo + pirfenidone (n=177)**
Cough	17 (21.8)	13 (17.1)	28 (16.1)	43 (24.3)
IPF	15 (19.2)	16 (21.1)	24 (13.8)	29 (16.4)
Dyspnoea	12 (15.4)	8 (10.5)	13 (7.5)	17 (9.6)
Fatigue	11 (14.1)	8 (10.5)	26 (14.9)	19 (10.7)
Nasopharyngitis	11 (14.1)	15 (19.7)	29 (16.7)	24 (13.6)
Upper respiratory tract infection	11 (14.1)	10 (13.2)	29 (16.7)	36 (20.3)
Bronchitis	10 (12.8)	7 (9.2)	17 (9.8)	11 (6.2)
Diarrhoea	8 (10.3)	10 (13.2)	17 (9.8)	19 (10.7)
Dizziness	8 (10.3)	9 (11.8)	7 (4.0)	11 (6.2)
Headache	7 (9.0)	9 (11.8)	10 (5.7)	15 (8.5)
Arthralgia	5 (6.4)	8 (10.5)	10 (5.7)	7 (4.0)
Nausea	7 (9.0)	4 (5.3)	22 (12.6)	18 (10.2)
Vascular disorders	4 (5.1)	7 (9.2)	19 (10.9)	13 (7.3)
Photosensitivity reaction	2 (2.6)	1 (1.3)	6 (3.4)	22 (12.4)
Rash	6 (7.7)	4 (5.3)	16 (9.2)	20 (11.3)
Decreased appetite	3 (3.8)	3 (3.9)	15 (8.6)	18 (10.2)

AE: adverse event; IPF: idiopathic pulmonary fibrosis.

SUPPLEMENTAL TABLE S7. Summary of AEs by preferred terms in ≥10% of patients in either arm of Cohort A during the lebrikizumab exposure period[#]

**AE**	**Lebrikizumab (N=130)**
Any	126 (96.9)
IPF	31 (23.8)
Cough	28 (21.5)
Nasopharyngitis	27 (20.8)
Diarrhoea	24 (18.5)
Bronchitis	22 (16.9)
Upper respiratory tract infection	21 (16.2)
Dyspnoea	20 (15.4)
Fatigue	19 (14.6)
Constipation	17 (13.1)
Dizziness	17 (13.1)
Back pain	15 (11.5)
Nausea	15 (11.5)
Urinary tract infection	15 (11.5)
Lower respiratory tract infection	14 (10.8)

AE: adverse event; IPF: idiopathic pulmonary fibrosis.

[#] The lebrikizumab exposure period included all adverse events reported from the first dose of study drug until the end of safety follow-up. This included patients who were randomised to receive lebrikizumab and all patients who received lebrikizumab during the open-label treatment period (including patients who were randomised to receive placebo).

SUPPLEMENTAL TABLE S8. Summary of causes of death

Deaths, n (%)	Cohort A			Cohort B		
	**Lebrikizumab**	**Placebo**	**All patients**	**Lebrikizumab + pirfenidone**	**Placebo + pirfenidone**	**All patients**
Total, at any time	15	4	19	11	18	29
Placebo-controlled period	**(n=78)**	**(n=76)**	**(N=154)**	**(n=174)**	**(n=177)**	**(N=351)**
All causes[#]	3 (3.8)	3 (3.9)	6 (3.9)	9 (5.2)	13 (7.3)	22 (6.3)
IPF	2	1	3	5	8[¶]	13
Pneumonia	0	0	0	1	1	2
Pulmonary embolism	1[¶]	0	1	0	0	0
Acute respiratory failure	0	1	1	0	1	1
Sudden death	0	1	1	0	0	0
Graft vs. host disease	0	0	0	1	0	1
Septic shock	0	0	0	1	0	1
Cardiovascular accident	0	0	0	1	0	1
Dyspnoea	0	0	0	0	1	1
Pulmonary fibrosis	0	0	0	0	1	1
Respiratory failure	0	0	0	0	1	1
Lebrikizumab exposure period[+]	**(N=130)**					
All causes, at any time	15 (11.5)	–	–	–	–	–
During placebo-controlled period (above)	3[§]	–	–	–	–	–
During open-label lebrikizumab	9	–	9	–	–	–
IPF	4	–	4	–	–	–
IPF and hypoxia	1	–	1	–	–	–
Acute myocardial infarction	1	–	1	–	–	–

Malignant lung neoplasm	1	–	1	–	–	–
Lower respiratory tract infection	1	–	1	–	–	–
Acute respiratory failure	1[¶]	–	1[¶]	–	–	–
During safety follow-up (below)	3[§]	–	–	–	–	–
Safety follow-up						
All causes	3[f]	1	4	2	5	7
IPF	2	1[¶]	3	0	4	4
Pleural effusion	1	0	1	0	1	1
Cardiac failure	0	0	0	2	0	2

[#] Including deaths that occurred ≤30 days after last dose of lebrikizumab; this count does not match the count of deaths for the efficacy analysis, which excluded deaths after lung transplant or after the 52-week placebo-controlled period.

[¶] Included 1 death that was assessed by the investigator to be treatment related.

[+] The lebrikizumab exposure period included all AEs reported from the first dose of study drug until the end of safety follow-up. This included patients who were randomised to receive lebrikizumab and all patients who received lebrikizumab during the open-label treatment period (including patients who were randomised to receive placebo).

[§] Of 15 deaths that occurred during the lebrikizumab exposure period, 3 were also counted during the placebo-controlled period (lebrikizumab arm) and 3 were also counted during safety follow-up.

[f] Included 2 patients in safety follow-up after double-blind lebrikizumab treatment and 1 after open-label lebrikizumab treatment after having received placebo.

SUPPLEMENTAL TABLE S9. List of investigators and ethics review approvals for each study site by country

Country	Investigator	Institutional review board/independent ethics committee name and address (if available)	Approval date
Australia	A. Glanville	SSWAHS Ethics Review Committee (RPAH Zone), c/- Research Development Office Level 3, Building 92, Missenden Road, 2050, Camperdown, NSW, Australia	08 Sep 2015
Australia	I. Glaspole	The Alfred Hospital Research Ethics Committee, The Alfred Hospital, 55 Commercial Road, 3004, Melbourne, VIC, Australia	17 Feb 2014
Australia	M. Phillips	Bellberry Human Research Ethics Committee, Bellberry Limited, 229 Greenhill Road, 5065, Dulwich, SA, Australia	16 Oct 2013
Australia	F. Thien	Northern Sydney Local Health District Human Research Ethics Committee, Level 13, Kolling Building, 2065, St. Leonards, NSW, Australia	28 Mar 2014
Australia	L. Troy	SSWAHS Ethics Review Committee (RPAH Zone), c/- Research Development Office Level 3, Building 92, Missenden Road, 2050, Camperdown, NSW, Australia	10 Apr 2014
Belgium	B. Bondue	Central EC, Commissie Medische Ethiek UZ KU Leuven, Campus Gasthuisberg E330 Herestraat 49, 3000 Leuven	28 Apr 2014
Belgium	C. Dahlqvist	Central EC, Commissie Medische Ethiek UZ KU Leuven, Campus Gasthuisberg E330 Herestraat 49, 3000 Leuven	28 Apr 2014
Belgium	T. Pieters	Central EC, Commissie Medische Ethiek UZ KU Leuven, Campus Gasthuisberg E330 Herestraat 49, 3000 Leuven	28 Apr 2014
Belgium	W. Wuyts	Central EC, Commissie Medische Ethiek UZ KU Leuven, Campus Gasthuisberg E330 Herestraat 49, 3000 Leuven	28 Apr 2014
Canada	N. Khalil	University of British Columbia Office of Research Services, 102-6190 Agronomy Road, Vancouver, BC Canada V6T 1Z3	25 Nov 2013
Canada	M. Mura	Western University Research Support Services Bldg., Rm. 5150, London, ON, Canada N6G 1G9	1 Nov 2013
Canada	S. Provencher	Institut Universitaire de Cardiologie et de Pneumologie de Quebec 2725 chemin Sainte-Foy, Local 2614, QC, Canada G1V 4G5	28 Jan 2014
France	B. Crestani	CPP Ile de France I, Hotel Dieu, 1 Place du Parvis de Notre-Dame, 75181, Paris, France	17 Sep 2013
France	S. Jouneau	CPP Ile de France I, Hotel Dieu, 1 Place du Parvis de Notre-Dame, 75181, Paris, France	17 Sep 2013
France	H. Nunes	CPP Ile de France I, Hotel Dieu, 1 Place du Parvis de Notre-Dame, 75181, Paris, France	17 Sep 2013
France	B. Wallaert	CPP Ile de France I, Hotel Dieu, 1 Place du Parvis de Notre-Dame, 75181, Paris, France	17 Sep 2013
Germany	J. Behr	EK Essen Ethik-Kommission der Medizinischen Fakultät der Universität Duisburg-Essen, Robert-Koch-Str. 9-11, 45147 Essen, Germany	4 Nov 2013
Germany	U. Costabel	EK Essen Ethik-Kommission der Medizinischen Fakultät der Universität Duisburg-Essen, Robert-Koch-Str. 9-11, 45147 Essen, Germany	4 Nov 2013
Germany	A. Günther	EK Essen Ethik-Kommission der Medizinischen Fakultät der Universität Duisburg-Essen, Robert-Koch-Str. 9-11, 45147 Essen, Germany	4 Nov 2013
Germany	P. Hammerl	EK Essen Ethik-Kommission der Medizinischen Fakultät der Universität Duisburg-Essen, Robert-Koch-Str. 9-11, 45147 Essen, Germany	4 Nov 2013
Germany	A.-M. Kirsten	EK Essen Ethik-Kommission der Medizinischen Fakultät der Universität Duisburg-Essen, Robert-Koch-Str. 9-11, 45147 Essen, Germany	4 Nov 2013
Germany	M. Kreuter	EK Essen Ethik-Kommission der Medizinischen Fakultät der Universität Duisburg-Essen, Robert-Koch-Str. 9-11, 45147 Essen, Germany	4 Nov 2013
Italy	S. Harari	IRCCS Multimedica Sezione Comitato Etico Centrale IRCCS Regione Lombardia, Via Milanese 300, 20099 Sesto San Giovanni (MI) Italy	18 Feb 2014
Italy	P. Rottoli	Comitato Etico Area Vasta Sud, Est, viale Bracci 16, 53100, Siena, Toscana, Italy	19 Nov 2013
Italy	S. Tomassetti	Comitato Etico Di Area Vasta Romagna E Irst, Via Piero Maroncelli, 40, 47014 Meldola (FC), Italy	16 Apr 2014
Italy	C. Vancheri	Comitato Etico Catania 1, Azienda Ospedaliero, Universitaria “Policlinico - Vittorio Emanuele,” via S.Sofia, 78, 95123 Catania, Italy	10 Dec 2013

Country	Investigator	Institutional review board/independent ethics committee name and address (if available)	Approval date
Japan	Y. Inoue	Institutional Review Board of National Hospital Organization Kinki - Chuo Chest Medical Center, 1180, Nagasonecho, Kita-ku, Sakai-shi Osaka, Japan 591-8555	12 Dec 2014
Japan	Y. Kondoh	Tosei General Hospital IRB 160, Nishioiwakecho, Seto-shi, Aichi, Japan 489-8642	26 Nov 2014
Japan	T. Ogura	Kanagawa Cardiovascular and, Respiratory Center Institutional Review, Board, 6-16-1, Tomioka-higashi, Kanazawa-ku, Kanagawa, Yokohama, Japan 236-0051	10 Dec 2014
Mexico	U. Chavarria Martinez	Comité de Ética en Investigación de la Facultad de Medicina y Hospital Universitario de la UANL, Av. Francisco I Madero y Av. Gonzalitos S/N, Col. Mitras, Centro Monterrey Nuevo Leon, 64460, Monterrey Nuevo Leon, Mexico	20 Dec 2013
Mexico	A. Ramirez	CEI Unidad de Investigacion Clinica en Medicina S.C; Comite de Etica en Investigacion, Av. La clinica No. 2520 - 522 y 524, Col. Sertoma, 64718, Monterrey, Nuevo Leon, Mexico	8 Oct 2013
Mexico	M. Selman Lama	CEI INER Ismael Cosio; Comite de Etica en Investigacion, Instituto Nacional de Enfermedades Respiratorias, Calz. de Tlalpan 4502 Del. Tlalpan, Col. Seccion XVI, Ciudad de México. C.P. 14080, Mexico	19 Feb 2014
Peru	S. Castro	Asociación Benefica Prisma EC, Av. Carlos Gonzales 251, Maranga, L-32, Lima, Peru	15 Nov 2013
Peru	A. G. Guerreros Benavides	Asociación Benefica Prisma EC, Carlos Gonzales 251, San Miguel 15088, Lima, Peru	22 Jan 2015
Peru	A. Matsuno	Asociación Benefica Prisma EC, Av. Carlos Gonzales 251, Maranga, L-32, Lima, Peru	23 Oct 2013
Poland	H. Batura-Gabryel	Komisja Bioetyki Uniwersytetu Medycznego w Lodzi, Al. Kosciuszki 4, 90-, 419, Lodz, Poland	11 Feb 2014
Poland	J. Kus	Komisja Bioetyki Uniwersytetu Medycznego w Lodzi, Al. Kosciuszki 4, 90-, 419, Lodz, Poland	11 Mar 2014
Poland	W. Piotrowski	Komisja Bioetyki Uniwersytetu Medycznego w Lodzi, Al. Kosciuszki 4, 90-, 419, Lodz, Poland	24 Sep 2013
Spain	J. Ancochea Bermudez	Hospital Universitario la Princesa; Comité, Etico de Investigacion Clinica, C/ Diego de Leon, 62, 28006, Madrid, Spain	16 Sep 2013
Spain	E. Fernandez-Fabrellas	CEIC Hospital General Universitario de Valencia, Avda. Tres Cruces s/n, 46014, Valencia, Spain	16 Sep 2013
Spain	M. Molina Molina	CEIC Hospital de Bellvitge, Carrer de la Feixa Llarga, s/n, 08907 L'Hospitalet de Llobregat, Barcelona, Spain	20 Sep 2013
Spain	A. Nieto Barbero	CEIC Hospital Clinico San Carlos, Farmacologia/1 planta Norte. Puerta G., Professor Martin Lagos s/n, 28040, Madrid, Spain	12 Feb 2014
Spain	J. A. Rodriguez-Portal	CEIC Hospital de Bellvitge, Carrer de la Feixa Llarga, s/n, 08907 L'Hospitalet de Llobregat, Barcelona, Spain	16 Dec, 2013
United Kingdom	H. Adamali	Southmead Hospital; Respiratory Department; Research & Innovation, Southmead Road, Westbury-on-Trym, Bristol, BS10 5NB, UK	31 Jan 2014
United Kingdom	P. Beirne	London - Hampstead Research Ethics Committee, Barlow House 3rd Floor, 4 Minshull Street Manchester, M1 3DZ, UK	31 Jan 2014
United Kingdom	Z. Borrill	London - Hampstead Research Ethics Committee, Barlow House 3rd Floor, 4 Minshull Street Manchester, M1 3DZ, UK	6 Nov 2013
United Kingdom	S. Fletcher	Southampton General Hospital; R&D (Trust), R&D office, Mailpoint 138, Duthie building (trust), Tremona Road, Southampton, SO16 6YD, UK	6 Nov 2013
United Kingdom	T. Maher	London - Hampstead Research Ethics Committee, Barlow House 3rd Floor, 4 Minshull Street Manchester, M1 3DZ, UK	6 Nov 2013
United Kingdom	H. Parfrey	Papworth Hospital NHS Foundation Trust; Respiratory Department; Research and Development office, Papworth Everard, Cambridge, CB23 3RE, UK	21 Mar 2014
United Kingdom	L. Spencer	London - Hampstead Research Ethics Committee, Barlow House 3rd Floor, 4 Minshull Street Manchester, M1 3DZ, UK	6 Nov 2013
United States	D. Antin-Ozerkis	Yale University IRB, 150 Munson St., 3rd Floor, P.O. Box 208327, New Haven CT, 06520-8327, USA	18 Oct 2013

Country	Investigator	Institutional review board/independent ethics committee name and address (if available)	Approval date
United States	A. Awab	University of Oklahoma IRB, 1000 Stanton L. Young Blvd, LIB176, Oklahoma City, OK 73117, USA	26 Jan 2014
United States	R. Bascom	Hershey Medical Center; Institutional Review Board, 600 Centerview Drive, Mail Code A115, Rm 1140, Hershey, PA, 17033, USA	17 Dec 2013
United States	N. Bhatt	Western International Review Board, 3535 Seventh Avenue SW, P.O. Box 12029, Olympia, WA, 98502-5010, USA	4 Nov 2013
United States	E. J. Britt	University of Maryland, Baltimore IRB 620 W Lexington St., Baltimore, MD 21201, USA	24 Sep 2013
United States	A. H. Case	Piedmont Healthcare, 1968 Peachtree Road, NW Atlanta, Georgia 30309, USA	11 Nov 2013
United States	C. Daniels	Mayo Clinic IRB, 201 Building, Room 4-60, 200 First St. SW, Rochester, MN 55905, USA	14 Mar 2014
United States	J. A. De Andrade	Western International Review Board, 3535 Seventh Avenue SW, P.O. Box 12029, Olympia, WA, 98502-5010, USA	1 Oct 2013
United States	K. DeBoer	Western International Review Board, 3535 Seventh Avenue SW, P.O. Box 12029, Olympia, WA, 98502-5010, USA	17 Jul 2013
United States	T. DeMarini	Western International Review Board, 3535 Seventh Avenue SW, P.O. Box 12029, Olympia, WA, 98502-5010, USA	22 Jul 2014
United States	D. Dilling	Loyola University Chicago Health Sciences Division IRB, 2160, S First Ave., Maywood, IL, 60153, USA	2 Oct 2013
United States	J. Dorf	Western International Review Board, 3535 Seventh Avenue SW, P.O. Box 12029, Olympia, WA, 98502-5010, USA	23 Apr 2014
United States	N. Ettinger	St. Luke’s Hospital IRB Office 2732, 4401 Wornall Road, Kansas City, MO 64111, USA	4 Sep 2013
United States	J. Ferguson	Western International Review Board, 3535 Seventh Avenue SW, P.O. Box 12029, Olympia, WA, 98502-5010, USA	30 Jul 2013
United States	A. K. Gerke	Western International Review Board, 3535 Seventh Avenue SW, P.O. Box 12029, Olympia, WA, 98502-5010, USA	10 Jan 2014
United States	K. Gibson	WIRB, 1019 39th Avenue SE, Suite 120, Puyallup, WA, 98374-2115, USA	24 Oct 2013
United States	M. Glassberg	University of Miami Human Subjects Research Office (M809), P.O. Box 016980, 1500 NW 12 Avenue, Suite 1002, Miami, Florida 33136, USA	5 Nov 2013
United States	J. A. Golden	University of California, San Francisco IRB Human Research Protection Program, Box 09623333 California Street, Suite 315, San Francisco, CA 94143, USA	7 Jun 2015
United States	N. Gupta	Western International Review Board, 3535 Seventh Avenue SW, P.O. Box 12029, Olympia, WA, 98502-5010, USA	1 Oct 2013
United States	T. Haddad	Western International Review Board, 3535 Seventh Avenue SW, P.O. Box 12029, Olympia, WA, 98502-5010, USA	6 Jun 2014
United States	M. Hamblin	University of Kansas Medical Center/Human Subjects Committee, MS-1032, 3901 Rainbow Blvd., Kansas City, KS, 66160, USA	17 Dec 2013
United States	C. Himanshu	Western International Review Board, 3535 Seventh Avenue SW, P.O. Box 12029, Olympia, WA, 98502-5010, USA	26 Mar 2014
United States	M. Horton	Johns Hopkins Medicine IRB1620 McElderry St., Reed Hall - B130, Baltimore, MD 21205-1911, USA	5 May 2014
United States	J. T. Huggins	Medical University of South Carolina IRB, 19 Hagood Avenue, Suite 601, MSC857, Charleston, SC 29425, USA	11 Feb 2014
United States	M. C. Kallay	Western International Review Board, 3535 Seventh Avenue SW, P.O. Box 12029, Olympia, WA, 98502-5010, USA	31 Dec 2013
United States	R. Kaner	Weill Cornell Medicine IRB, 1300 York Avenue, Box 89, New York, NY 10065, USA	18 Oct 2013
United States	H. Kim	University of Minnesota IRB, D528 Mayo Memorial Building, 420 Delaware Street SE, MMC, Minneapolis, MN 55455, USA	17 Sep 2014
United States	K. Knox	Western International Review Board, 3535 Seventh Avenue SW, P.O. Box 12029, Olympia, WA, 98502-5010, USA	27 Aug 2013
United States	L. H. Lancaster	Vanderbilt University IRB, 504 Oxford House, Nashville, TN 37232-4315, USA	2 Jan 2014
United States	A. Lee	Mayo Clinic IRB, 201 Building, Room 4-60, 200 First St. SW, Rochester, MN 55905, USA	13 Mar 2014

Country	Investigator	Institutional review board/independent ethics committee name and address (if available)	Approval date
United States	R. Lipchik	Medical College of Wisconsin IRB MFRC 3040, MACC Fund Research Center Office of Research, 8701 Watertown Plank Road, Milwaukee, WI 53226-0509, USA	27 Aug 2014
United States	N. Marchetti	Temple University IRB, 1801 N Broad Street, Conwell Hall, Room 401, Philadephia, PA 19122, USA	14 Apr 2014
United States	K. Meyer	Western International Review Board, 3535 Seventh Avenue SW, P.O. Box 12029, Olympia, WA, 98502-5010, USA	14 Mar 2014
United States	A. Nambiar	UT Health Science Center, San Antonio, IRB, Mail code 7830, 7703 Floyd Curl Drive, San Antonio, TX 78229-3900, USA	28 Aug 2013
United States	I. Noth	The University of Chicago IRB, 5841 S Maryland Ave., I-625 MC 7132, Chicago, IL 60637, USA	18 Oct 2013
United States	M. Padilla	Mount Sinai Health System, One Gustave L. Levy Place, Box 1081, New York, NY 10029-6574, USA	17 Mar 2014
United States	J. Palminteri	Chesapeake IRB, 6940 Columbia Gateway Drive, Suite 110, Columbia, MD 21046, USA	3 Feb 2014
United States	S. Puthalapattu	Southern Arizona VA Health Care System IRB, 3601 S 6th Ave., Mail Code (0-151), Bldg. 77, Tucson, AZ 85723, USA	13 May 2014
United States	Z. Safdar	Baylor College of Medicine; Institutional Review Board, One Baylor Plaza, 600D, Houston, TX 77030, USA	23 Jan 2014
United States	M. B. Scholand	Institutional Review Board for Research with Human Subjects, University of Utah, Research Administration Building, 75 S 2000 E, Salt Lake City, UT 84112, USA	16 Dec 2013
United States	B. Shea	Lifespan, Research Protection Office, CORO West, Suite 1.300, One Hoppin Street, Providence, RI 02903, USA	26 Jul 2013
United States	O. Shlobin	Western International Review Board, 3535 Seventh Avenue SW, P.O. Box 12029, Olympia, WA, 98502-5010, USA	13 Mar 2014
United States	W. Strauss	Western International Review Board, 3535 Seventh Avenue, SW, P.O. Box 12029, Olympia, WA, 98502-5010, USA	18 Jul 2013
United States	J. Swigris	Western International Review Board, 3535 Seventh Avenue, SW, P.O. Box 12029, Olympia, WA, 98502-5010, USA	1 Aug 2013
United States	A. Thompson	University of Nebraska Medical Center Academic and Research Services Building 3000, 987830 Nebraska Medical Center, Omaha, NE 68198-7830, USA	2 Jun 2014
United States	M. L. Wencel	Via Christi Hospitals Wichita, Inc. IRB, 1100 N St. Francis, Suite 300, Wichita, KS 67214, USA	12 Jul 2013

SUPPLEMENTAL TABLE S10. Patients receiving antifibrotics as rescue therapy in Cohort A during the placebo-controlled period

Patients Receiving Antifibrotic Therapy, n (%)	Cohort A		
	Lebrikizumab (n=78)	Placebo (n=76)	All patients (N=154)
Pirfenidone	13 (16.7)	8 (10.5)	21 (13.6)
Nintedanib	1 (1.3)	3 (3.9)	4 (2.6)
