# Supplementary material for: Phase 2 trial to assess lebrikizumab in patients with idiopathic pulmonary fibrosis
Source: Eur Respir J. 2021 Feb 4;57(2):1902442. doi: 10.1183/13993003.02442-2019 (PMC7859504; doi:10.1183/13993003.02442-2019)
Supplement: Supplementary file 2 [file ERJ-02442-2019.Shareable.pdf]

ORIGINAL ARTICLE
INTERSTITIAL LUNG DISEASE

SHAREABLE PDF

# Phase 2 trial to assess lebrikizumab in patients with idiopathic pulmonary fibrosis

Toby M. Maher[1], Ulrich Costabel [2], Marilyn K. Glassberg [3], Yasuhiro Kondoh[4], Takashi Ogura[5], Mary Beth Scholand [6], David Kardatzke[7], Monet Howard[7], Julie Olsson[7], Margaret Neighbors[7], Paula Belloni[7] and Jeffrey J. Swigris[8]

**Affiliations**: [1]NIHR Respiratory Clinical Research Facility, Royal Brompton Hospital and National Heart and Lung Institute, Imperial College London, London, UK. [2]Ruhrlandklinik, University of Duisburg-Essen, Essen, Germany. [3]University of Arizona, College of Medicine, Phoenix, AZ, USA. [4]Tosei General Hospital, Aichi, Japan. [5]Kanagawa Cardiovascular and Respiratory Center, Yokohama, Japan. [6]University of Utah, Salt Lake City, UT, USA. [7]Genentech, Inc., South San Francisco, CA, USA. [8]National Jewish Health, Denver, CO, USA.

**Correspondence**: Toby M. Maher, Fibrosis Research Group, National Heart and Lung Institute, Imperial College of London, Sir Alexander Fleming Building, London SW7 2AZ, UK. E-mail: t.maher@imperial.ac.uk

@ERSpublications
**This phase 2 RCT found no benefit in FVC decline over 52 weeks in IPF patients for lebrikizumab *versus* placebo as monotherapy (n=78 *versus* 76) or in combination with pirfenidone (n=174 *versus* 177); pirfenidone therapy was consistent with previous results** https://bit.ly/313NVR8

**Cite this article as:** Maher TM, Costabel U, Glassberg MK, *et al.* Phase 2 trial to assess lebrikizumab in patients with idiopathic pulmonary fibrosis. *Eur Respir J* 2021; 57: 1902442 [https://doi.org/10.1183/13993003.02442-2019].

This single-page version can be shared freely online.

ABSTRACT This phase 2, randomised, double-blind, placebo-controlled trial evaluated the efficacy and safety of lebrikizumab, an interleukin (IL)-13 monoclonal antibody, alone or with background pirfenidone therapy, in patients with idiopathic pulmonary fibrosis (IPF).

Patients with IPF aged ⩾40 years with forced vital capacity (FVC) of 40%–100% predicted and diffusing capacity for carbon monoxide of 25%–90% predicted and who were treatment-naïve (cohort A) or receiving pirfenidone (2403 mg·day$^{-1}$; cohort B) were randomised 1:1 to receive lebrikizumab 250 mg or placebo subcutaneously every 4 weeks. The primary endpoint was annualised rate of FVC % predicted decline over 52 weeks.

In cohort A, 154 patients were randomised to receive lebrikizumab (n=78) or placebo (n=76). In cohort B, 351 patients receiving pirfenidone were randomised to receive lebrikizumab (n=174) or placebo (n=177). Baseline demographics were balanced across treatment arms in both cohorts. The primary endpoint (annualised rate of FVC % predicted decline) was not met in cohort A (lebrikizumab *versus* placebo, −5.2% *versus* −6.2%; p=0.456) or cohort B (lebrikizumab *versus* placebo, −5.5% *versus* −6.0%; p=0.557). In cohort B, a non-statistically significant imbalance in mortality favouring combination therapy was observed (hazard ratio 0.42 (95% CI 0.17–1.04)). Pharmacodynamic biomarkers indicated lebrikizumab activity. The safety profile was consistent with that in previous studies of lebrikizumab and pirfenidone as monotherapies.

Lebrikizumab alone or with pirfenidone was not associated with reduced FVC % predicted decline over 52 weeks despite evidence of pharmacodynamic activity. Lebrikizumab was well tolerated with a favourable safety profile. These findings suggest that blocking IL-13 may not be sufficient to achieve a lung function benefit in patients with IPF.

Copyright ©ERS 2021. This version is distributed under the terms of the Creative Commons Attribution Licence 4.0.
